# Sleep Problems Alter Proximal Risk of Negative Self‐Perceptions on Suicide Risk

**DOI:** 10.1111/sltb.70092

**Published:** 2026-03-31

**Authors:** Jennifer J. Muehlenkamp, Connor M. O'Brien, Ross Jacobucci, Brooke A. Ammerman

**Affiliations:** ^1^ Department of Psychology Hibbard Humanities Hall 277 University of Wisconsin‐Eau Claire Eau Claire Wisconsin USA; ^2^ Department of Psychology University of Wisconsin‐Madison Madison Wisconsin USA; ^3^ Center for Healthy Minds University of Wisconsin‐Madison Madison Wisconsin USA

**Keywords:** burdensomeness, ecological momentary assessment, EMA, self‐harm, self‐hate, sleep, suicide

## Abstract

**Introduction:**

Perceiving oneself as a burden and self‐hate are core drivers of suicidal thoughts and behaviors. Sleep problems also contribute to elevated risk for suicide. These factors are often studied in isolation and the impact of sleep problems on the risk conferred by negative self‐perceptions to suicide is unclear. The current study evaluated how sleep problems interact with state‐ and trait‐level self‐hate and perceived burdensomeness in predicting suicide intent.

**Methods:**

Data came from 25 adult outpatients with recent suicide ideation who completed a 21‐day EMA protocol including three daily prompts assessing prior night hours slept, sleep problems, burdensomeness, self‐hate, and suicide urges (*n* observations = 1092). Bayesian multilevel cumulative ordinal models with random intercepts were run.

**Results:**

All variables except sleep hours had significant within‐person effects on suicide urges. There were significant negative interactions between within‐person burdensomeness and between‐person sleep problems, as well as a within‐person self‐hate and between‐person sleep problems. Among those with more sleep problems, the association between burdensomeness or self‐hate and suicide urges was less positive, suggesting sleep problems may override the risk‐related effects of negative self‐perceptions.

**Conclusion:**

These findings underscore the complexity of suicide risk and suggest that interventions focusing on improving sleep may reduce near‐term risk for suicide.

## 1 Introduction

Suicide remains a major public health concern, yet the field continues to struggle with identifying the short‐term psychological processes that contribute to acute risk (Centers for Disease Control and Prevention 2024a, 2024b). Two constructs consistently implicated as risk factors for suicide ideation (SI), planning, and behaviors are sleep disturbances (Porras‐Segovia et al. 2019; Rössler et al. 2018) and negative self‐perceptions (Brott and Veilleux 2024; O'Neill et al. 2021). These factors have largely been examined in isolation and little is known about their synchronic impact on short‐term suicide risk. Thus, this study aims to investigate the conjoint effects of sleep and negative self‐perceptions on momentary suicidal urges with intent.

Prior cross‐sectional research has suggested that sleep deprivation (Baiden et al. 2020; Joseph et al. 2023) and poorer sleep quality (Batterham et al. 2021; Bozzay et al. 2025) are associated with an increased likelihood of suicidal thoughts and behaviors (STBs). Moreover, longitudinal research has established a bidirectional relationship between poor sleep quality and suicide risk over the course of 1 and 2 years, (Liu et al. 2024), and 30‐years (Rössler et al. 2018). Examining these effects at the proximal level, researchers found subjective reports of a poor night's sleep and shorter sleep duration were associated with within‐person, next‐day SI and intent (Brüdern et al. 2022; Kivelä et al. 2024; Rogers and Bozzay 2024). These findings have also been replicated using smartwatch‐based estimates of sleep quality (Cox et al. 2023; Littlewood et al. 2018). Despite progress in understanding the near‐term link between sleep and suicide risk, most studies have focused on general suicide ideation resulting in a gap in understanding more severe, intent‐laden urges that may represent a closer indicator of acute risk and most fail to consider sleep in conjunction with other key risk factors such as negative self‐perceptions.

Theoretical models of suicidal behavior emphasize negative self‐perceptions (e.g., burdensomeness, self‐hate) as part of a central belief system driving suicide urges and behaviors (Jobes 2023; Joiner 2005; Rudd and Bryan 2021). Cross‐sectional studies frequently identify perceiving oneself as a burden to be a salient predictor of the intensity of SI (Duffy et al. 2020; Gill et al. 2023) and longitudinal studies have found that perceived burdensomeness predicts SI both 3 days (Crosby et al. 2020) and 4 months (Roeder and Cole 2019) later. Ecological momentary assessment (EMA) studies have also underscored perceived burdensomeness as a salient, proximal predictor of SI within the same‐ and next‐day (Hutchinson et al. 2025; Jacobucci et al. 2023; Kivelä et al. 2024; Shou et al. 2025). Moreover, interventions targeting perceived burdensomeness have been successful at reducing SI (Lieberman et al. 2023), lending validity to the connection.

Despite perceived burdensomeness being a likely near‐term risk factor, it is still unknown under what conditions its influence may be strongest. Notably, prior literature has found that higher levels of perceived burdensomeness may be positively correlated with more severe insomnia symptoms (Nadorff et al. 2014) and may mediate the relationship with SI (Simmons et al. 2024). Further, those with more severe insomnia symptoms reported higher perceived burdensomeness following a social exclusion experimental paradigm (Chu et al. 2019). While some research has failed to find significant associations between clinical insomnia and perceived burdensomeness (Silva et al. 2015), there has been limited work extending findings to a proximal risk framework.

Although the Interpersonal Theory of Suicide (ITS; Joiner 2005) conceptualizes perceived burdensomeness as arising from two components, self‐liability and self‐hatred, it is increasingly recognized that self‐hatred may function as a distinct, proximal affective state with relevance to suicide risk beyond its role within perceived burdensomeness (Sorgi‐Wilson et al. 2023; Turnell et al. 2019). Perceived burdensomeness reflects a cognitive interpersonal appraisal that one's existence is a burden to others, whereas self‐hatred captures a more global, affective, inwardly directed negative self‐evaluation that may be particularly relevant to immediate SI risk (Bentley et al. 2021; Szeto et al. 2023; Zullo et al. 2022). Thus, while related, these constructs may fluctuate differently and have unique proximal effects or interactions with sleep problems. Examining self‐hate independently from, but alongside, perceived burdensomeness could provide insight into whether this affective component contributes unique momentary risk even when the broader construct of perceived burdensomeness remains constant.

Qualitative analyses of patient responses show that individuals frequently describe self‐hate as a primary driver of suicidal urges, independent of interpersonal beliefs such as perceived burdensomeness (Lynch et al. 2024; Madsen and Harris 2021). Yet, studies of self‐hate specifically have been sparse and limited to cross‐sectional designs. Higher trait self‐criticism, a related but less severe form of self‐hate, has been linked to SI cross‐sectionally (O'Neill et al. 2021), within an experimental vignette paradigm (Brott and Veilleux 2023), and prospectively across 1 month (Bentley et al. 2021), 3 months (O'Connor and Noyce 2008), and 5 months (Campos et al. 2013). However, to our knowledge, such associations have yet to be examined proximally and the contextual factors, such as sleep quality, potentially impacting these relationships are understudied. Research has linked poor sleep to elevated trait self‐criticism (Bar et al. 2020; Norton et al. 2023; Teixeira et al. 2016), and one study found higher levels of self‐criticism prospectively predicted poorer sleep quality across a 45‐day period (Bar et al. 2020). However, studies have not examined sleep's effect on proximal self‐hate nor the subsequent influence on short‐term suicide risk.

There is evidence suggesting that poor sleep, perceived burdensomeness, and self‐hate may intersect in predicting suicide urges with intent. Yet, a significant gap exists in evaluating these constructs simultaneously, within the short term, and in relation to intent‐laden suicide urges that may be a closer proxy for imminent behavioral risk (Jobes and Joiner 2019). The current study addresses these gaps by using a 21‐day EMA design to examine how prior night sleep problems interact with momentary perceived burdensomeness and self‐hate to predict suicide urges with intent. Although perceived burdensomeness includes self‐hatred as one of its theorized components, we treat perceived burdensomeness and self‐hate as distinct predictors because they represent different cognitive and affective psychological processes (Turnell et al. 2019; Zullo et al. 2022), and therefore may have unique proximal effects. We hypothesized that (A) sleep problems would predict stronger next‐day suicide urges; (B) perceived burdensomeness would show a positive association with suicide urges; (C) self‐hate would also show a positive association with suicide urges; and (D) sleep problems would moderate these associations, such that greater sleep difficulties will strengthen these associations.

## 2 Method

### 2.1 Participants

Participants included 25 adults (*M*_age_ = 35.6, SD = 14.36) receiving outpatient behavioral health treatment with elevated scores (≥ 2) on the PHQ‐9 suicide item. Females comprised slightly more than half the sample (*n* = 15, 60%), 12% identified as queer, and 44.8% were in a committed relationship. Almost all participants identified as white (96%), with 8.3% identifying with Hispanic ethnicity. Four participants (16.0%) identified as a Veteran, and 22 (88%) identified as heterosexual. A quarter (25%) reported having graduated college, 70.8% graduated high school/GED, and 4.2% had not completed high school/GED. Half the sample (54.5%) reported an annual income under $50,000. Based upon medical chart review, 21 (84%) had attempted suicide in their lifetime and 4 (16%) had a suicide attempt within the past year. Ten participants (40%) had a past‐year psychiatric emergency department visit and seven (28%) had an inpatient stay over 2 days in the past year. Participants had an average of 3.24 (SD = 1.33) diagnoses. All participants had current diagnoses of both major depressive disorder and an anxiety disorder; 36% also had a diagnosis of post‐traumatic stress disorder, 32% borderline personality disorder, 28% bipolar disorders, 16% attention deficit hyperactivity disorder, 8% eating disorder, and 8% obsessive‐compulsive disorder. The final analytic sample included 1092 observations for concurrent (same‐day) analyses and 628–647 observations for prospective (next‐day) analyses, depending on the specific model.

### 2.2 Procedures

Potentially eligible participants were identified through electronic medical record audits of recent visit responses on the Patient Health Questionnaire (Kroenke and Spitzer 2002; inclusion criteria: ≥ 2 on PHQ‐9 item 9; no psychosis; had a behavioral health provider at the clinic). Eligible participants were then contacted by study staff through email and/or phone and invited to attend an enrollment session (*n* = 29) where they were provided informed consent, completed baseline assessments, and were oriented to the EMA protocol. Study staff assisted participants with downloading and using the mEMA app (by Ilumivu) on their personal smartphone; all participants completed a practice EMA prompt. Prior to leaving the first session, participants were screened for imminent suicide risk (none screened positive) and provided information about local crisis services. For safety monitoring during data collection, the EMA app notified clinical research staff via email if a high‐risk response was entered for the suicide urge item. Staff followed up by phone to complete a risk screening as soon as possible and no longer than 10 h after notification (Nock et al. 2021). One participant withdrew during the baseline assessment, one participant was deemed ineligible at the enrollment visit due to current psychotic symptoms, and two participants withdrew during the first week of the study due to frustrations with the EMA app, resulting in a final sample of 25 participants. Ethics approval was obtained from the hospital system IRB where data was collected.

EMA data collection began after the study enrollment session and continued for 21 days. Participants received notifications three times daily that were randomized within 4‐h time blocks in the morning (7 am–10 am), afternoon (1 pm–4 pm), and evening (6 pm–9 pm), with a 60‐min response window before the survey closed. Participants were compensated for their baseline visit ($30) and up to $150 using a prorated incentive scale based upon their EMA survey completion rate.

### 2.3 EMA Survey Items

During each morning assessment window, participants completed items inquiring about their recent night's sleep. Participants were asked to estimate the number of hours spent sleeping using a sliding bar response option ranging from 0 to 12+ h. A single item based on the Insomnia Severity Index (Bastien et al. 2001), was used to evaluate subjective sleep quality/problems: *How severe were your sleep problems last night (e.g., trouble falling or staying asleep, waking up too early)*. Response options ranged from 0 = none/no difficulties to 8 = very severe problems/difficulties. At each EMA assessment, participants rated their current urge to attempt suicide using an item based on published EMA studies of suicide (e.g., Forkmann et al. 2018; Kleiman et al. 2017), “Right now, how strong is your urge to kill yourself?” Response options ranged from 0 = none to 8 = extremely strong. Perceived burdensomeness was assessed with a single item, “Right now, how much do you feel like a burden on others?” utilizing a scale ranging from 0 = no burdensomeness to 8 = high/severe burdensomeness. Similarly, self‐hate was assessed with the item, “Right now, how much self‐hate do you feel?” along a scale where 0 = no self‐hate to 8 = high/severe self‐hate.

### 2.4 Data Analysis

To examine within‐person and between‐person effects, two versions of each variable were generated: a person‐centered and a grand mean‐centered version, respectively. For prospective analyses, lead (*t* + 1) outcome variables were created, with models restricted to assessments occurring within 10 h of each other to ensure temporal proximity (omitting across‐day effects). Bayesian multilevel cumulative ordinal models were fitted using the brms package (Bürkner 2017) in R. Model selection via leave‐one‐out cross‐validation confirmed that cumulative ordinal models provided superior fit compared to Poisson or zero‐inflated Poisson alternatives.

All models included random intercepts for participants to account for the nested data structure. Models were estimated using Hamiltonian Monte Carlo with 4 chains of 1500 iterations each (750 warmup, 750 sampling), yielding 3000 posterior samples. Convergence was assessed via R‐hat values (all < 1.01) and effective sample sizes (Vehtari et al. 2021). The analysis tested: (1) main effects of sleep variables on concurrent and next‐assessment suicide urges, (2) main effects of perceived burdensomeness and self‐hate, and (3) interaction effects between sleep and self‐perceptions. For prospective models, autoregressive effects were controlled by including current suicide urges as a predictor. Statistical significance was determined using 95% credible intervals for main effects and 99% credible intervals for interaction effects to adjust for multiple testing, with effects considered significant when intervals did not contain zero.

## 3 Results

There were significant between‐person (estimate = 1.62, SE = 0.46, 99% CI = 0.72, 2.57) and within‐person (estimate = 0.16, SE = 0.06, 99% CI = 0.03, 0.28) effects of prior night sleep problems, but not number of hours slept (see Table S1), on suicide urge such that having more sleep problems predicted higher suicide urges. There was also a positive, within‐person effect of perceived burdensomeness on suicide urge when controlling for prior night sleep problems and the number of hours slept (estimate = 0.70, SE = 0.07, 99% CI = 0.58, 0.83), but no significant between‐person effect (estimate = 1.36, SE = 0.75, 99% CI = −0.16, 2.85; see Table S2). There was a positive, within‐person effect of self‐hate on suicide urge when controlling for prior night sleep problems and the number of hours slept (estimate = 0.85, SE = 0.07, 99% CI = 0.71, 0.99), but no significant between‐person effect (estimate = 0.52, SE = 0.53, 99% CI = −0.56, 1.55; see Table S3).

**FIGURE 1:**
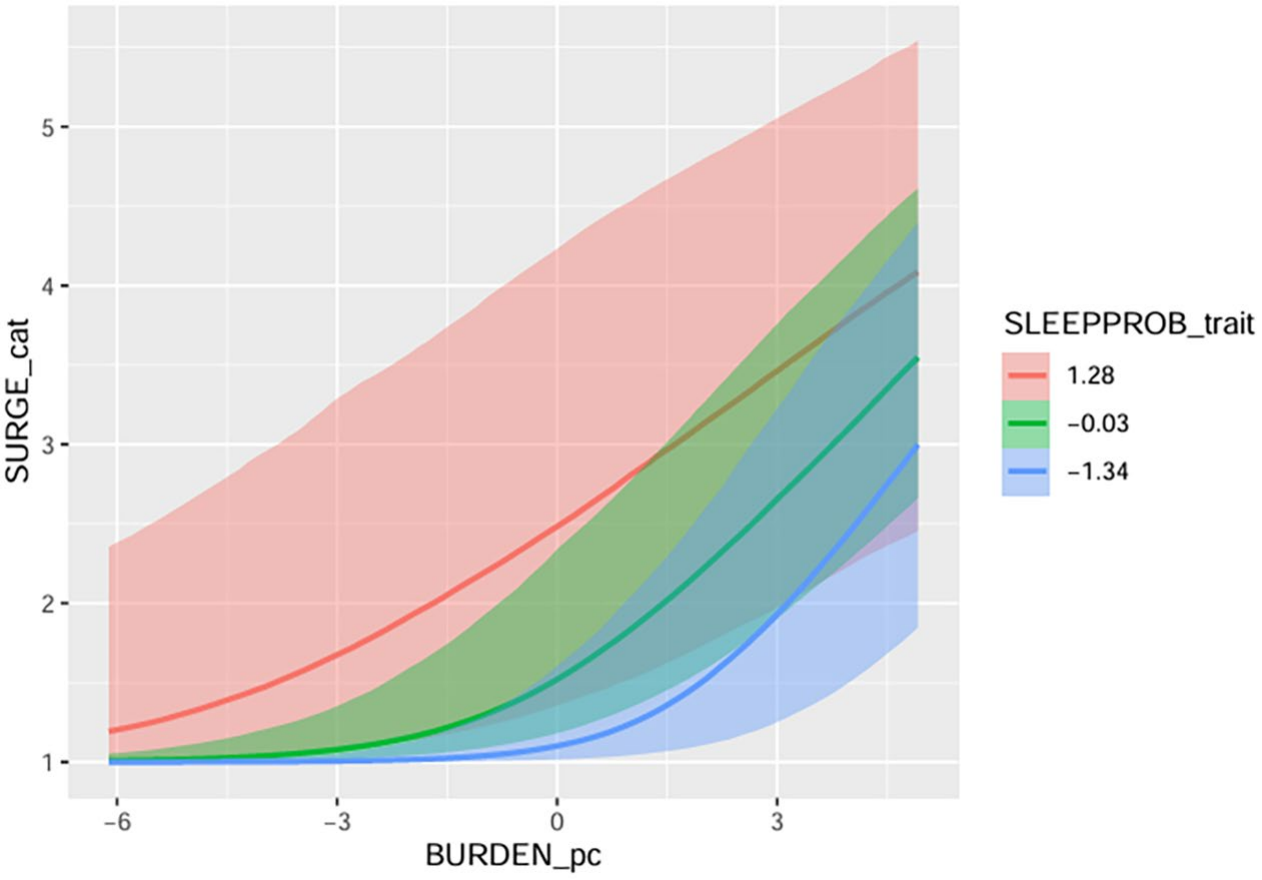
Interaction effect of sleep problems on burdensomeness in relation to suicide urges.

Full model results examining the interactions between sleep, burdensomeness, and self‐hate are reported in Tables 1 and 2, respectively. There was a significant interaction effect between within‐person burdensomeness and between‐person prior night sleep problems on suicide urges (see Table 1). Specifically, there was a weaker association between within‐person burdensomeness and suicide urges among those with more prior night sleep problems (see Figure 1). There were no significant interactions for models of between‐person burdensomeness and between‐person prior night sleep problems, nor any significant interactions for within‐person prior night sleep problems.

**TABLE 1:** Effects of perceived burdensomeness, sleep problems, and their interaction on suicide urge.

Variable	Estimate	SE	99% LCI	99% UCI	Rhat	Bulk ESS	Tail ESS
Intercept [1]	0.39	0.76	−2.29	2.15	1.00	773.00	960.00
Intercept [2]	2.05	0.76	−0.64	3.88	1.00	792.00	1022.00
Intercept [3]	3.45	0.77	0.80	5.28	1.00	800.00	943.00
Intercept [4]	4.81	0.79	1.96	6.63	1.00	843.00	1002.00
Intercept [5]	7.52	0.87	4.72	9.61	1.00	942.00	1023.00
Intercept [6]	9.50	0.92	6.61	11.73	1.00	1023.00	1025.00
PB (between person and person)	0.37	0.45	−0.76	1.78	1.00	1003.00	1046.00
PB (within person and person)	0.74	0.07	0.55	0.91	1.00	3356.00	2566.00
Sleep Prob (between person and person)	1.50	0.70	−0.30	3.33	1.00	794.00	1131.00
Sleep Prob (within person and person)	0.12	0.06	−0.02	0.27	1.00	3638.00	2335.00
PB (within person–person) × Sleep Prob (within person–person)	0.03	0.04	−0.08	0.14	1.00	3708.00	2259.00
PB (between person–person) × Sleep Prob (within person–person)	0.09	0.04	0.00	0.18	1.00	3299.00	2370.00
PB (within person–person) × Sleep Prob (between person–person)	−0.17	0.05	−0.30	−0.04	1.00	3924.00	2329.00
PB (between person–person) × Sleep Prob (between person–person)	0.15	0.31	−0.73	1.00	1.10	847.00	1184.00

Abbreviations: CI, confidence interval; ESS, effective sample size; PB, perceived burdensomeness; SE, standard error; Sleep Prob, sleep problems.

**TABLE 2:** Effects of self‐hate, sleep problems, and their interaction on suicide urge.

Variable	Estimate	SE	99% LCI	99% UCI	Rhat	Bulk ESS	Tail ESS
Intercept [1]	0.85	0.74	−1.23	2.74	1.00	795.00	1134.00
Intercept [2]	2.65	0.75	0.56	4.60	1.00	830.00	1180.00
Intercept [3]	4.12	0.76	2.0	6.09	1.00	860.00	1204.00
Intercept [4]	5.50	0.78	3.35	7.52	1.00	890.00	1325.00
Intercept [5]	8.21	0.85	5.94	10.31	1.01	998.00	1178.00
Intercept [6]	10.16	0.90	7.63	12.49	1.00	1086.00	1279.00
SH (between person and person)	0.71	0.56	−0.76	2.33	1.01	1097.00	1324.00
SH (within person and person)	0.93	0.08	0.75	1.13	1.00	3582.00	2263.00
Sleep Prob (between person and person)	1.07	0.73	−0.93	2.94	1.00	1019.00	1079.00
Sleep Prob (within person–person)	0.06	0.06	−0.09	0.22	1.00	3509.00	2222.00
SH (between person and person) × Sleep Prob (between person and person)	0.45	0.33	−0.40	1.39	1.00	1300.00	1629.00
SH (between person and person) × Sleep Prob (within person–person)	0.08	0.05	−0.03	0.20	1.00	4201.00	2464.00
SH (within person–person) × Sleep Prob (between person and person)	−0.25	0.05	−0.38	−0.11	1.00	4102.00	2376.00
SH (within person–person) × Sleep Prob (within person–person)	0.03	0.04	−0.08	0.14	1.10	4039.00	2349.00

Abbreviations: CI, confidence interval; ESS, effective sample size; SE, standard error; SH, self‐hate; Sleep Prob, sleep problems.

**FIGURE 2:**
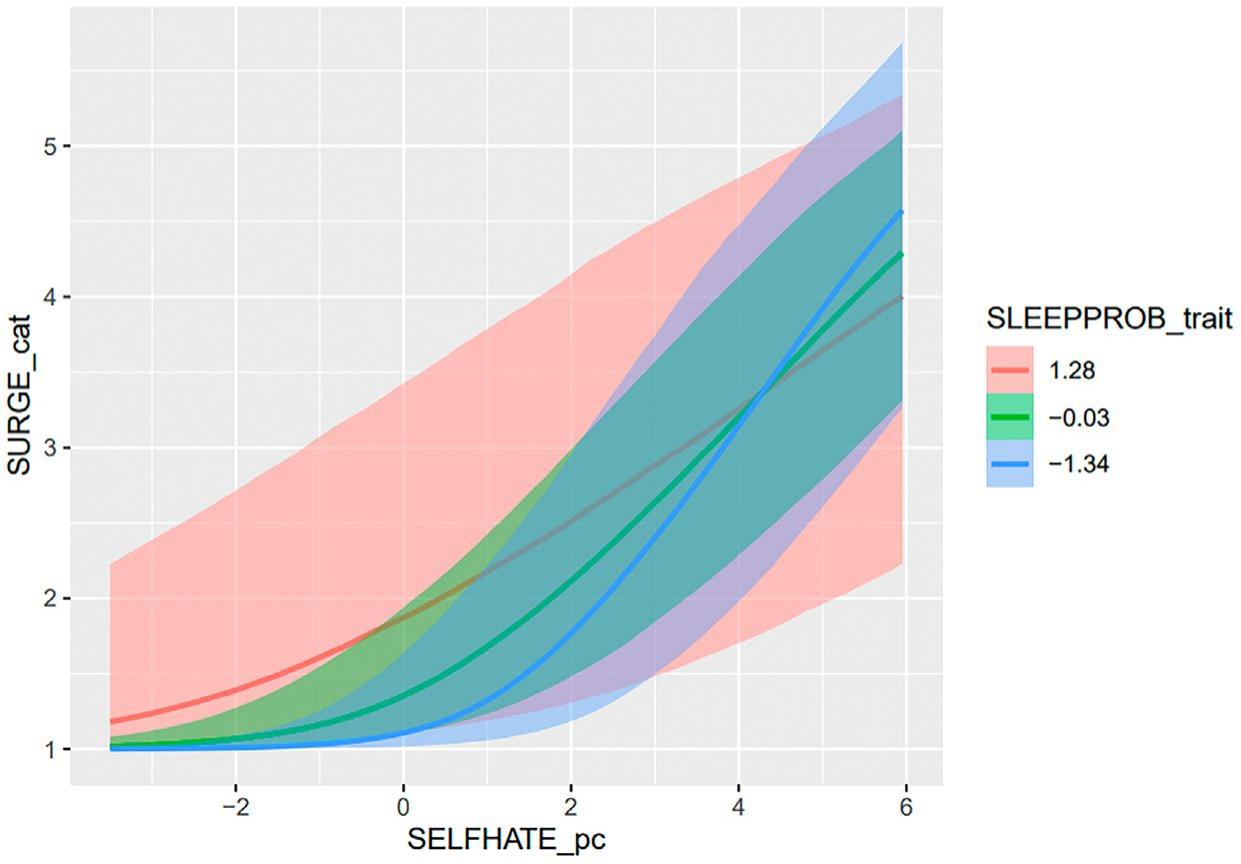
Interaction effect of sleep problems on self‐hate in relation to suicide urges.

A similar pattern emerged between sleep and self‐hate (see Table 2). There was a significant interaction effect for within‐person self‐hate and between‐person prior night sleep problems on suicide urges, such that there was a weaker association between within‐person self‐hate and suicide urges among those with more prior night sleep problems (see Figure 2). There were no significant interactions for between‐person self‐hate and between‐person prior night sleep problems, nor any significant interactions for within‐person prior night sleep problems.

## 4 Discussion

The current study fills a gap in the existing literature by examining within‐ and between‐person effects and interactions among sleep problems, perceived burdensomeness, and self‐hate on proximal suicide urges, with study hypotheses being partially supported. Both within‐ and between‐person prior night subjective sleep problems were associated with increased next‐day suicide urges, supporting prior work suggesting that sleep disturbances contribute to heightened suicide risk (Bozzay et al. 2025; Cox et al. 2023; Liu et al. 2024). Further, we found that within‐person, but not between‐person, variations in perceived burdensomeness and self‐hate were each significantly related to suicide urges, with elevations from one's average corresponding with stronger concurrent urges. These findings replicate prior studies (e.g., Hutchinson et al. 2025, Szeto et al. 2023) and provide evidence that fluctuations in sleep, burdensomeness, and self‐hate are associated with short‐term risk for suicide, warranting clinical attention in suicide risk assessments and interventions. Of note, the number of hours spent sleeping were unrelated to suicide urges, but both within‐ and between‐person perceived sleep difficulties were, suggesting that perceived quality of sleep may be more salient to suicide risk than objective sleep markers such as total sleep time (see also Batterham et al. 2021; Oakey‐Frost et al. 2026; Romier et al. 2023). The lack of between‐person effects for self‐hate and burdensomeness also underscores the importance of client‐centered suicide risk assessments as changes within a client's experience of self‐hate and/or burdensomeness may be the indication of escalating or declining suicide risk rather than the presence of greater or lesser levels of these constructs relative to other clients.

In addition to the direct effects on suicide urges, there were significant interactions with between‐person sleep problems and both within‐person perceived burdensomeness and self‐hate. Contrary to expectations, the interactions were negative, suggesting that the proximal risk relationship between suicide urges, burdensomeness and self‐hate are weakened for those who report greater prior night sleep problems. Given that sleep problems were proximally associated with stronger suicide urges both within‐ and between‐persons in the current sample, it appears that sleep problems may overpower the risk conferred by negative self‐perceptions. Sleep problems are known to disrupt many biological and psychological processes implicated in suicide risk, including dysregulation within the prefrontal cortex and stress response systems (Kearns et al. 2020; Palagini et al. 2022) which may temporarily overwhelm a person's agency to cope, driving short‐term increased suicide urges regardless of other risk factors. This idea is further supported by a recent longitudinal study of adults, finding that sleep problems prospectively predicted suicide attempts above and beyond the effects of psychiatric disorders and other risk factors (Geoffroy et al. 2021). However, when sleep problems are low, other more insidious factors such as burdensomeness and self‐hate, and their fluctuations, may become the salient factors exerting an effect on suicidal urges. Additional studies are needed to understand and tease apart the interactive effects of sleep and psychological risk processes on short‐term suicide risk. However, if the current results hold true, it suggests that clinicians may want to prioritize improving sleep quality (e.g., Bishop et al. 2020; Mournet and Kleiman 2024) before addressing negative self‐perceptions to decrease suicide risk among those reporting sleep difficulties.

These findings provide innovative insights into the possible way sleep problems may influence short‐term escalations in suicide risk but need to be considered in the context of the study limitations. While a strength of the study includes the participation of individuals from a high‐risk clinical sample with good study compliance (and observation) rate, the sample size is small and homogenous, restricting the generalizability of the results. Replications of the current work are needed within larger and more diverse samples. The use of single items to assess study constructs, while common practice to reduce participant burden within EMA studies (Song et al. 2023), is a limitation as it prevents a nuanced understanding of the variables and may be less likely to capture potential variations between or within persons. Our decision to model self‐hate and perceived burdensomeness separately may raise conceptual questions. The intent was to clarify whether the affective component (self‐hate) contributes unique proximal risk beyond the broader construct. The single item assessment of self‐hate and perceived burdensomeness precluded our ability to tease apart self‐hate from self‐liability, so future studies using multi‐item measures could more precisely disentangle the unique effects of these components. Due to the short time frame of the study and low base‐rate of suicide we were unable to examine behaviors, although suicide urges with intent represent a close proxy (Jobes and Joiner 2019; Stanley et al. 1992). Future studies should aim to examine a continuum of suicide thoughts and behaviors as outcomes to offer a more precise understanding of how sleep affects short‐term risk to better inform interventions.

The current study offers one of the first examinations of the interaction between short‐term changes in perceived sleep quality, burdensomeness, and self‐hate on proximal risk for suicide urges. Sleep problems are associated with increased vulnerability for next‐day suicide urges and appear to affect the way momentary negative self‐perceptions relate to proximal risk such that within‐person variations in burdensomeness and self‐hate have weaker associations to suicide urges when sleep problems are high. These findings further highlight the importance of sleep in the understanding of suicide risk (e.g., Baldini et al. 2024; Romier et al. 2023), underscoring the need to embed questions about sleep quality within suicide risk assessments, risk monitoring, and clinical interventions. Future studies are needed to clarify the complex effects of sleep disruptions on short‐term suicide risk processes.

## Author Contributions

**Jennifer J. Muehlenkamp:** conceptualization, methodology, investigation, data curation, writing – original draft, writing – review and editing, project administration, funding acquisition; **Connor M. O'Brien:** writing – original draft, writing – review and editing; **Ross Jacobucci:** formal analysis, software, writing – original draft; **Brooke A. Ammerman:** writing – review and editing, formal analysis – support.

## Funding

This work was supported by Mayo Clinic Health System/UW‐Eau Claire Research Innovation Council.

## Disclosure

No AI or AI‐assisted technologies were used in the writing of this manuscript.

## Ethics Statement

All methods and procedures associated with this study were approved by the ethics board/Internal Review Board of the hospital clinic where the data collection occurred. And all participants provided written and verbal informed consent prior to participation.

## Conflicts of Interest

The auhtors declare no conflicts of interest.

## Supporting information


**Table S1:** Effects of prior night sleep problems and hours slept on suicide urge.
**Table S2:** Effects of perceived burdensomeness, prior night sleep problems, and hours slept on suicide urge.
**Table S3:** Effects of self‐hate, prior night sleep problems, and hours slept on suicide urge.

## Data Availability

Per company regulations regarding patient research data, the data for this study is available upon direct, reasonable request to the corresponding author.

## References

[sltb70092-bib-0001] Baiden, P. , S. K. Tadeo , B. C. Tonui , J. D. Seastrunk , and G. O. Boateng . 2020. “Association Between Insufficient Sleep and Suicidal Ideation Among Adolescents.” Psychiatry Research 287: 112579. 10.1016/j.psychres.2019.112579. 31627959

[sltb70092-bib-0002] Baldini, V. , M. Gnazzo , G. Rapelli , et al. 2024. “Association Between Sleep Disturbances and Suicidal Behavior in Adolescents: A Systematic Review and Meta‐Analysis.” Frontiers in Psychiatry 15: 1341686. 39421072 10.3389/fpsyt.2024.1341686 PMC11483864

[sltb70092-bib-0003] Bar, M. , G. Schrieber , N. Gueron‐Sela , G. Shahar , and L. Tikotzky . 2020. “Role of Self‐Criticism, Anxiety, and Depressive Symptoms in Young Adults' Insomnia.” International Journal of Cognitive Therapy 13, no. 1: 15–29. 10.1007/s41811-019-00058-2.

[sltb70092-bib-0066] Bastien, C. H. , A. Vallières , and C. M. Morin . 2001. “Validation of the Insomnia Severity Index as an Outcome Measure for Insomnia Research.” Sleep Medicine 2, no. 4: 297–307. 11438246 10.1016/s1389-9457(00)00065-4

[sltb70092-bib-0004] Batterham, P. J. , A. Werner‐Seidler , A. L. Calear , S. McCallum , and A. Gulliver . 2021. “Specific Aspects of Sleep Disturbance Associated With Suicidal Thoughts and Attempts.” Journal of Affective Disorders 282: 574–579. 33440302 10.1016/j.jad.2020.12.150

[sltb70092-bib-0005] Bentley, K. H. , D. L. Coppersmith , E. M. Kleiman , et al. 2021. “Do Patterns and Types of Negative Affect During Hospitalization Predict Short‐Term Post‐Discharge Suicidal Thoughts and Behaviors?” Affective Science 2, no. 4: 484–494. 35465415 10.1007/s42761-021-00058-6 PMC9022604

[sltb70092-bib-0071] Bishop, T. M. , P. G. Walsh , L. Ashrafioun , J. E. Lavigne , and W. R. Pigeon . 2020. “Sleep, Suicide Behaviors, and the Protective Role of Sleep Medicine.” Sleep Medicine 66: 264–270. 31727433 10.1016/j.sleep.2019.07.016

[sltb70092-bib-0051] Bozzay, M. L. , G. T. Wallace , and M. L. Rogers . 2025. “Sleep Quality and Disruptive Nocturnal Behaviors as Short‐Term Predictors of Suicidal Intent: An Ecological Momentary Assessment Study.” Journal of Psychiatric Research 181: 304–311. 39637723 10.1016/j.jpsychires.2024.11.066 PMC11750597

[sltb70092-bib-0006] Brott, K. H. , and J. C. Veilleux . 2023. “Examining State Self‐Criticism and Self‐Efficacy as Factors Underlying Hopelessness and Suicidal Ideation.” Suicide and Life‐Threatening Behavior 54, no. 2: 207–220. 10.1111/sltb.13034. 38112324

[sltb70092-bib-0072] Brott, K. H. , and J. C. Veilleux . 2024. “Examining State Self‐Criticism and Self‐Efficacy as Factors Underlying Hopelessness and Suicidal Ideation.” Suicide & Life‐Threatening Behavior 54, no. 2: 207–220. 38112324 10.1111/sltb.13034

[sltb70092-bib-0008] Brüdern, J. , N. Hallensleben , I. Höller , et al. 2022. “Sleep Disturbances Predict Active Suicidal Ideation the Next Day: An Ecological Momentary Assessment Study.” BMC Psychiatry 22, no. 1: 65. 10.1186/s12888-022-03716-6. 35086519 PMC8793208

[sltb70092-bib-0073] Bürkner, P. C. 2017. “brms: An R Package for Bayesian Multilevel Models Using Stan.” Journal of Statistical Software 80: 1–28.

[sltb70092-bib-0010] Campos, R. C. , A. Besser , and S. J. Blatt . 2013. “Recollections of Parental Rejection, Self‐Criticism and Depression in Suicidality.” Archives of Suicide Research 17, no. 1: 58–74. 10.1080/13811118.2013.748416. 23387404

[sltb70092-bib-0011] Centers for Disease Control and Prevention . 2024a. Facts About Suicide. Centers for Disease Control and Prevention.

[sltb70092-bib-0012] Centers for Disease Control and Prevention . 2024b. Suicide Data and Statistics. Centers for Disease Control and Prevention. https://www.cdc.gov/suicide/facts/data.html?CDC_AAref_Val=https%3A%2F%2Fwww.cdc.gov%2Fsuicide%2Fsuicide‐data‐statistics.html.

[sltb70092-bib-0014] Chu, C. , M. A. Hom , J. K. Hirsch , and T. E. Joiner . 2019. “Thwarted Belongingness and Perceived Burdensomeness Explain the Relationship Between Sleep Problems and Suicide Risk Among Adults Identifying as Sexual and/or Gender Minorities.” Psychology of Sexual Orientation and Gender Diversity 6, no. 1: 22–33. 10.1037/sgd0000301.

[sltb70092-bib-0015] Cox, R. C. , S. L. Brown , B. N. Chalmers , and L. N. Scott . 2023. “Examining Sleep Disturbance Components as Near‐Term Predictors of Suicide Ideation in Daily Life.” Psychiatry Research 326: 115323. 37392522 10.1016/j.psychres.2023.115323 PMC10527974

[sltb70092-bib-0016] Crosby, E. S. , K. L. Zuromski , and T. K. Witte . 2020. “Perceived Burdensomeness Is a Curvilinear, Short‐Term Predictor of Suicide Ideation in a Community Sample of Adults.” Suicide and Life‐Threatening Behavior 50, no. 6: 1205–1213. 10.1111/sltb.12716. 33098120

[sltb70092-bib-0017] Duffy, M. E. , N. E. Mueller , J. R. Cougle , and T. E. Joiner . 2020. “Perceived Burdensomeness Uniquely Accounts for Suicidal Ideation Severity in Social Anxiety Disorder.” Journal of Affective Disorders 266: 43–48. 10.1016/j.jad.2020.01.116. 32056911

[sltb70092-bib-0067] Forkmann, T. , L. Spangenberg , D. Rath , et al. 2018. “Assessing Suicidality in Real Time: A Psychometric Evaluation of Self‐Report Items for the Assessment of Suicidal Ideation and its Proximal Risk Factors Using Ecological Momentary Assessments.” Journal of Abnormal Psychology 127, no. 8: 758–769. 30299116 10.1037/abn0000381

[sltb70092-bib-0018] Geoffroy, P. A. , M. A. Oquendo , P. Courtet , et al. 2021. “Sleep Complaints Are Associated With Increased Suicide Risk Independently of Psychiatric Disorders: Results From a National 3‐Year Prospective Study.” Molecular Psychiatry 26, no. 6: 2126–2136. 32355334 10.1038/s41380-020-0735-3

[sltb70092-bib-0058] Gill, P. R. , M. Arena , C. Rainbow , et al. 2023. “Social Connectedness and Suicidal Ideation: The Roles of Perceived Burdensomeness and Thwarted Belongingness in the Distress to Suicidal Ideation Pathway.” BMC Psychology 11, no. 1: 312. 37803474 10.1186/s40359-023-01338-5 PMC10557190

[sltb70092-bib-0021] Hutchinson, E. , L. Scott , S. Choukas‐Bradley , and J. Silk . 2025. “Interpersonal Risk Factors for Suicide in Daily Life Among Young People: A Review of Intensive Longitudinal Studies.” Development and Psychopathology 37: 1–21. 10.1017/S0954579424001810 39743871

[sltb70092-bib-0022] Jacobucci, R. , K. McClure , and B. A. Ammerman . 2023. “Comparing the Role of Perceived Burdensomeness and Thwarted Belongingness in Prospectively Predicting Active Suicidal Ideation.” Suicide and Life‐Threatening Behavior 53, no. 2: 198–206. 10.1111/sltb.12933. 36458583

[sltb70092-bib-0055] Jobes, D. A. 2023. Managing Suicidal Risk: A Collaborative Approach. Guilford Publications.

[sltb70092-bib-0023] Jobes, D. A. , and T. E. Joiner . 2019. “Reflections on Suicidal Ideation [Editorial].” Crisis: The Journal of Crisis Intervention and Suicide Prevention 40, no. 4: 227–230. 10.1027/0227-5910/a000615. 31274031

[sltb70092-bib-0057] Joiner, T. 2005. Why People Die by Suicide. Harvard University Press.

[sltb70092-bib-0024] Joseph, V. A. , N. T. Kreski , and K. M. Keyes . 2023. “Sleep Deprivation and Suicide Risk Among Minoritized US Adolescents.” BMC Psychiatry 23, no. 1: 638. 10.1186/s12888-023-05074-3. 37653474 PMC10472686

[sltb70092-bib-0069] Kearns, J. C. , D. D. Coppersmith , A. C. Santee , C. Insel , W. R. Pigeon , and C. R. Glenn . 2020. “Sleep Problems and Suicide Risk in Youth: A Systematic Review, Developmental Framework, and Implications for Hospital Treatment.” General Hospital Psychiatry 63: 141–151. 30301558 10.1016/j.genhosppsych.2018.09.011

[sltb70092-bib-0053] Kivelä, L. M. , W. Van der Does , and N. Antypa . 2024. “Sleep, Hopelessness, and Suicidal Ideation: An Ecological Momentary Assessment and Actigraphy Study.” Journal of Psychiatric Research 177: 46–52. 38972264 10.1016/j.jpsychires.2024.06.039

[sltb70092-bib-0059] Kleiman, E. M. , B. J. Turner , S. Fedor , E. E. Beale , J. C. Huffman , and M. K. Nock . 2017. “Examination of Real‐Time Fluctuations in Suicidal Ideation and Its Risk Factors: Results From Two Ecological Momentary Assessment Studies.” Journal of Abnormal Psychology 126, no. 6: 726. 28481571 10.1037/abn0000273

[sltb70092-bib-0064] Kroenke, K. , and R. L. Spitzer . 2002. “The PHQ‐9: A New Depression Diagnostic and Severity Measure.” Psychiatric Annals 32, no. 9: 509–515.

[sltb70092-bib-0025] Lieberman, A. , A. R. Gai , M. L. Rogers , et al. 2023. “Targeting Perceived Burdensomeness to Reduce Suicide Risk.” Behavior Therapy 54, no. 4: 696–707. 10.1016/j.beth.2022.12.002. 37330258

[sltb70092-bib-0027] Littlewood, D. L. , S. D. Kyle , L.‐A. Carter , S. Peters , D. Pratt , and P. Gooding . 2018. “Short Sleep Duration and Poor Sleep Quality Predict Next‐Day Suicidal Ideation: An Ecological Momentary Assessment Study.” Psychological Medicine 49, no. 3: 403–411. 10.1017/s0033291718001009. 29697037 PMC6331731

[sltb70092-bib-0028] Liu, X. , Y. Yang , Z.‐Z. Liu , and C.‐X. Jia . 2024. “Bidirectional Associations Between Sleep Problems and Suicidal Thought/Attempt in Adolescents: A 3‐Wave Data Path Analysis.” Journal of Affective Disorders 350: 983–990. 10.1016/j.jad.2024.01.153. 38244795

[sltb70092-bib-0062] Lynch, T. , V. C. Bathe , and D. A. Jobes . 2024. “The Content of Patient‐Identified Suicidal Drivers Within CAMS Treatment Planning.” Archives of Suicide Research 28, no. 1: 411–417. 36550770 10.1080/13811118.2022.2151958

[sltb70092-bib-0063] Madsen, J. , and K. M. Harris . 2021. “Negative Self‐Appraisal: Personal Reasons for Dying as Indicators of Suicidality.” PLoS One 16, no. 2: e0246341. 33529221 10.1371/journal.pone.0246341 PMC7853472

[sltb70092-bib-0029] Mournet, A. M. , and E. M. Kleiman . 2024. “A Systematic Review and Meta‐Analysis on the Efficacy of Sleep Interventions to Treat Suicidal Ideation.” Journal of Sleep Research 33, no. 4: e14133. 38164094 10.1111/jsr.14133

[sltb70092-bib-0030] Nadorff, M. R. , M. D. Anestis , S. Nazem , H. Claire Harris , and E. Samuel Winer . 2014. “Sleep Disorders and the Interpersonal–Psychological Theory of Suicide: Independent Pathways to Suicidality?” Journal of Affective Disorders 152: 505–512. 10.1016/j.jad.2013.10.011. 24182416

[sltb70092-bib-0065] Nock, M. K. , E. M. Kleiman , M. Abraham , et al. 2021. “Consensus Statement on Ethical & Safety Practices for Conducting Digital Monitoring Studies With People at Risk of Suicide and Related Behaviors.” Psychiatric Research and Clinical Practice 3, no. 2: 57–66. 34414359 10.1176/appi.prcp.20200029 PMC8372411

[sltb70092-bib-0031] Norton, D. W. , O. Modesto , J. M. Bennett , and M. I. Fraser . 2023. “Sleep Disturbance Mediates the Link Between Both Self‐Compassion and Self‐Criticism and Psychological Distress During Prolonged Periods of Stress.” Applied Psychology. Health and Well‐Being 16, no. 1: 119–137. 10.1111/aphw.12474. 37501499

[sltb70092-bib-0068] Oakey‐Frost, N. , E. H. Moscardini , M. L. Rogers , and J. J. Muehlenkamp . 2026. “Comparing Objective Measures of Sleep Disturbance and Sleep Related Impairments as Proximal Risk Indicators of Suicidal Intent and Non‐Suicidal Self‐Injury.” *Suicide and Life‐Threatening Behavior*. in press. 10.1111/sltb.70091 PMC13036479 41914013

[sltb70092-bib-0032] O'Connor, R. C. , and R. Noyce . 2008. “Personality and Cognitive Processes: Self‐Criticism and Different Types of Rumination as Predictors of Suicidal Ideation.” Behaviour Research and Therapy 46, no. 3: 392–401. 10.1016/j.brat.2008.01.007. 18308293

[sltb70092-bib-0033] O'Neill, C. , D. Pratt , M. Kilshaw , K. Ward , J. Kelly , and G. Haddock . 2021. “The Relationship Between Self‐Criticism and Suicide Probability.” Clinical Psychology & Psychotherapy 28, no. 6: 1445–1456. 10.1002/cpp.2593. 33847028

[sltb70092-bib-0070] Palagini, L. , P. A. Geoffroy , M. Miniati , et al. 2022. “Insomnia, Sleep Loss, and Circadian Sleep Disturbances in Mood Disorders: A Pathway Toward Neurodegeneration and Neuroprogression?” A Theoretical Review. CNS spectrums 27, no. 3: 298–308. 33427150 10.1017/S1092852921000018

[sltb70092-bib-0036] Porras‐Segovia, A. , M. M. Pérez‐Rodríguez , P. López‐Esteban , et al. 2019. “Contribution of Sleep Deprivation to Suicidal Behaviour: A Systematic Review.” Sleep Medicine Reviews 44: 37–47. 10.1016/j.smrv.2018.12.005. 30640161

[sltb70092-bib-0038] Roeder, K. M. , and D. A. Cole . 2019. “Simultaneous Longitudinal Examination of Hopelessness, Thwarted Belongingness, and Perceived Burdensomeness as Predictors of Suicide Ideation.” Suicide and Life‐Threatening Behavior 49, no. 4: 1058–1071. 10.1111/sltb.12508. 30099767

[sltb70092-bib-0052] Rogers, M. L. , and M. L. Bozzay . 2024. “Daily and Cumulative Sleep Duration as Predictors of Suicidal Desire and Intent: An Ecological Momentary Assessment Study.” Journal of Clinical Psychiatry 85, no. 2: 23m15164. 10.4088/JCP.23m15164 38836860

[sltb70092-bib-0039] Romier, A. , J. Maruani , J. Lopez‐Castroman , et al. 2023. “Objective Sleep Markers of Suicidal Behaviors in Patients With Psychiatric Disorders: A Systematic Review and Meta‐Analysis.” Sleep Medicine Reviews 68: 101760. 36706699 10.1016/j.smrv.2023.101760

[sltb70092-bib-0040] Rössler, W. , J. Angst , V. Ajdacic‐Gross , et al. 2018. “Sleep Disturbances and Suicidality–a Longitudinal Analysis From a Representative Community Study Over 30 Years.” Frontiers in Psychiatry 9: 320. 10.3389/fpsyt.2018.00320. 30061849 PMC6054984

[sltb70092-bib-0056] Rudd, M. D. , and C. J. Bryan . 2021. “The Brief Suicide Cognitions Scale: Development and Clinical Application.” Frontiers in Psychiatry 12: 737393. 34594254 10.3389/fpsyt.2021.737393 PMC8476787

[sltb70092-bib-0041] Shou, Y. , M. Gendi , R. Borschmann , et al. 2025. “Longitudinal Causal Dynamics Between Perceived Burdensomeness and Suicidal Ideation: Population‐Based Cohort Study.” Journal of Psychiatric Research 189: 388–394. 40580610 10.1016/j.jpsychires.2025.06.022

[sltb70092-bib-0042] Silva, C. , J. D. Ribeiro , and T. E. Joiner . 2015. “Mental Disorders and Thwarted Belongingness, Perceived Burdensomeness, and Acquired Capability for Suicide.” Psychiatry Research 226, no. 1: 316–327. 10.1016/j.psychres.2015.01.008. 25650048

[sltb70092-bib-0043] Simmons, Z. , S. Baldwin , J. Jones , et al. 2024. “Connecting the Dots Between Insomnia and Suicidal Ideation: The Mediating Roles of Depression, Emotion Dysregulation and Thwarted Belonging.” Research Directions: Sleep Psychology 1: e10.

[sltb70092-bib-0044] Song, J. , E. Howe , J. R. Oltmanns , and A. J. Fisher . 2023. “Examining the Concurrent and Predictive Validity of Single Items in Ecological Momentary Assessments.” Assessment 30, no. 5: 1662–1671. 36004406 10.1177/10731911221113563 PMC10248304

[sltb70092-bib-0060] Sorgi‐Wilson, K. M. , J. C. Cheung , N. K. Ciesinski , and M. S. McCloskey . 2023. “Cognition and Non‐Suicidal Self‐Injury: Exploring Relationships With Psychological Functions.” Archives of Suicide Research 27, no. 3: 1002–1018. 35924878 10.1080/13811118.2022.2106919 PMC9898468

[sltb70092-bib-0045] Stanley, B. , R. Winchel , A. Molcho , D. Simeon , and M. Stanley . 1992. “Suicide and the Self‐Harm Continuum: Phenomenological and Biochemical Evidence.” International Review of Psychiatry 4, no. 2: 149–155.

[sltb70092-bib-0061] Szeto, E. H. , E. Ammendola , A. Starkey , J. Hay , J. G. McClung , and C. J. Bryan . 2023. “Differences in Guilt, Shame, Self‐Anger, and Suicide Cognitions Based on Recent Suicide Ideation and Lifetime Suicide Attempt History.” Journal of Nervous and Mental Disease 211, no. 3: 226–232. 36166283 10.1097/NMD.0000000000001592

[sltb70092-bib-0046] Teixeira, I. , S. Simões , M. Marques , H. Espírito‐Santo , and L. Lemos . 2016. “Self‐Criticism and Self‐Compassion Role in the Occurrence of Insomnia on College Students.” European Psychiatry 33, no. S1: s268. 10.1016/j.eurpsy.2016.01.702.

[sltb70092-bib-0054] Turnell, A. I. , D. B. Fassnacht , P. J. Batterham , A. L. Calear , and M. Kyrios . 2019. “The Self‐Hate Scale: Development and Validation of a Brief Measure and its Relationship to Suicidal Ideation.” Journal of Affective Disorders 245: 779–787. 30448763 10.1016/j.jad.2018.11.047

[sltb70092-bib-0048] Vehtari, A. , A. Gelman , D. Simpson , B. Carpenter , and P. C. Bürkner . 2021. “Rank‐Normalization, Folding, and Localization: An Improved R for Assessing Convergence of MCMC (With Discussion).” Bayesian Analysis 16, no. 2: 667–718.

[sltb70092-bib-0050] Zullo, L. , H. Mbroh , A. Moorehead , S. C. Lee , B. D. Kennard , and S. M. Stewart . 2022. “Exploring the Construct of Perceived Burdensomeness Among Suicidal Adolescents: An Intervention Development Study.” Journal of Child and Family Studies 31, no. 7: 1994–2004.

