# Supplementary material for: Sleep Problems Alter Proximal Risk of Negative Self‐Perceptions on Suicide Risk
Source: Suicide Life Threat Behav. 2026 Mar 31;56(2):e70092. doi: 10.1111/sltb.70092 (PMC13036480; doi:10.1111/sltb.70092)
Supplement: Supplementary file 1 — Table S1: Effects of prior night sleep problems and hours slept on suicide urge. Table S2: Effects of perceived burdensomeness, prior night sleep problems, and hours slept on suicide urge. Table S3: Effects of self‐hate, prior night sleep problems, and hours slept on suicide urge. [file SLTB-56-0-s001.docx]

Supplemental Table 1. *Effects of Prior Night Sleep Problems and Hours Slept on Suicide Urge*

| **Variable** | **Estimate** | **SE** | **99%LCI** | **99%UCI** | **Rhat** | **Bulk ESS** | **Tail ESS** |
| --- | --- | --- | --- | --- | --- | --- | --- |
| Intercept [1] | 0.25 | 0.52 | -0.80 | 1.28 | 1.0 | 711.00 | 1148.00 |
| Intercept [2] | 1.73 | 0.52 | 0.68 | 2.80 | 1.0 | 706.00 | 1093.00 |
| Intercept [3] | 2.94 | 0.53 | 1.88 | 3.98 | 1.0 | 718.00 | 1154.00 |
| Intercept [4] | 4.14 | 0.55 | 3.02 | 5.20 | 1.0 | 755.00 | 1201.00 |
| Intercept [5] | 6.75 | 0.64 | 5.49 | 8.00 | 1.0 | 999.00 | 1622.00 |
| Intercept [6] | 8.66 | 0.70 | 7.29 | 10.06 | 1.0 | 1118.00 | 1695.00 |
| Hours Slept (within person-person) | -0.07 | 0.05 | -0.17 | 0.02 | 1.0 | 3438.00 | 2276.00 |
| Hours Slept (between person-person) | -0.23 | 0.61 | -1.44 | 1.10 | 1.0 | 775.00 | 1186.00 |
| Sleep Problems (between person-person) | 1.62 | 0.46 | 0.72 | 2.57 | 1.1 | 772.00 | 1353.00 |
| Sleep Problems (within person-person) | 0.16 | 0.06 | 0.03 | 0.28 | 1.0 | 3535.00 | 2463.00 |

*Note:* SE = standard error; LCI = lower confidence interval; UCI = upper confidence interval; ESS = effective sample size

Supplemental Table 2. *Effects of Perceived Burdensomeness, Prior Night Sleep Problems, and Hours Slept on Suicide Urge*

Supplemental Table 3. *Effects of Self-Hate, Prior Night Sleep Problems, and Hours slept on Suicide Urge*
